# T cell responses are elicited against Respiratory Syncytial Virus in the absence of signalling through TLRs, RLRs and IL-1R/IL-18R

**DOI:** 10.1038/srep18533

**Published:** 2015-12-21

**Authors:** Michelle Goritzka, Catherine Pereira, Spyridon Makris, Lydia R. Durant, Cecilia Johansson

**Affiliations:** 1 Centre for Respiratory Infections, Respiratory Infections Section, St Mary’s campus, National Heart and Lung Institute, Faculty of Medicine, Imperial College London, London, W2 1PG, UK

## Abstract

Pattern recognition receptors (PRRs) and cytokine receptors are key players in the initiation of immune responses to infection. PRRs detecting viral RNA, such as toll like receptor (TLR)-3, -7/8, and RIG-I like receptors (RLRs; RIG-I and MDA-5), as well as cytokine receptors such as interleukin 1 receptor (IL-1R), have been implicated in responses to RNA viruses that infect the airways. The latter includes respiratory syncytial virus (RSV), a human pathogen that can cause severe lower respiratory tract infections, especially in infants. To evaluate the collective contribution of PRRs and IL-1R signalling to RSV immunity, we generated *Myd88/Trif/Mavs*^−/−^ mice that are deficient in signalling by all TLRs, RLRs and IL-1R, as well as other cytokine receptors such as IL-18 receptor. Early production of pro-inflammatory mediators and lung infiltration by immune cells were completely abrogated in infected *Myd88/Trif/Mavs*^−/−^ mice. However, RSV-specific CD8^+^ T cells were elicited and recruited into the lungs and airways. Consistent with these findings, *Myd88/Trif/Mavs*^−/−^ mice survived RSV infection but displayed higher viral load and weight loss. These data highlight an unappreciated level of redundancy in pathways that couple innate virus sensing to adaptive immunity, providing the host with remarkable resilience to infection.

Respiratory syncytial virus (RSV) is a negative, single stranded RNA virus estimated to infect at least 37 million children worldwide every year. In most cases the symptoms manifest into a common cold. However, RSV can cause severe lower respiratory tract infection resulting in an estimated 200,000 deaths in children under the age of 5 every year^1^. The lack of vaccines against RSV and/or treatments for RSV infection emphasises the need to investigate the regulation of host immune responses to the virus.

CD4^+^ and CD8^+^ antiviral T cells are elicited during RSV infection in both mouse and human^2^,^3^. Although important for efficient viral clearance, in mouse models T cells have also been shown to contribute to RSV-associated immunopathology^4^. T cells are primed in the lung draining lymph nodes primarily by migrating lung dendritic cells (DCs)^5^,^6^ that acquired RSV antigens in the lung through direct infection or phagocytosis of dying virally-infected cells. Contact with viral pathogen-associated molecular patterns (PAMPs) facilitates the process by triggering pattern recognition receptors (PRRs) that signal to promote activation of DCs^7^,^8^. PRRs signalling activates transcription factors such as NF-κB, Jun and interferon regulatory factors (IRFs) family members, which not only induce DC activation but also lead to expression of type I interferons (IFN-α/β), other pro-inflammatory cytokines and chemokines by both DCs and other cell types, notably alveolar macrophages (AMs)^9^. The actions of PRRs across multiple cell types are largely responsible for early T cell-independent innate immune resistance to infection. PRRs implicated in recognition of RNA viruses such as RSV include members of the toll-like receptor (TLR) family that signal through the MyD88 adaptor (TLR2, 4, 7 and 8) or through the TRIF adaptor (TLR3 and 4)^10^. RNA viruses are additionally recognised by PRRs of the RLR family such as RIG-I and MDA5 as well as NOD2, a distinct PRR normally involved in responses to bacteria, which all signal via the adaptor MAVS^11^,^12^. Finally, in the context of influenza virus infection, DC activation can be driven by signalling through the IL-1 receptor (IL-1R) rather than PRRs^13^. Signalling through the IL-1R and the IL-18 receptor (IL-18R), as for most TLRs, require the adaptor MyD88^14^.

TLR2/6, TLR3, TLR4, TLR7, NOD2, RIG-I and MDA5 have all been implicated in RSV recognition^7^,^15^ and loss of function of some of them (or of their adaptors) in mice can impact innate immunity to the virus^9^,^11^,^16^,^17^,^18^,^19^,^20^,^21^,^22^. It is less clear to what extent these receptors are necessary for DC activation and priming of RSV-specific T cells. It has been reported that normal anti-RSV T cell responses can be elicited in *Mavs*^−/−^ mice or mice doubly-deficient in MyD88 and MAVS^9^,^21^,^22^. This might suggest that TLR3 and TLR4 signalling through TRIF can compensate for loss of other TLRs and of RLRs, indicative of a high level of redundancy in the immune response to RSV. We set out to test this hypothesis by creating mice deficient in all three adaptor proteins; MyD88, TRIF and MAVS. Here, we show that after RSV infection these *Myd88/Trif/Mavs*^−/−^ mice lose more weight and have higher viral load than control mice. The early inflammatory response to RSV is absent in *Myd88/Trif/Mavs*^−/−^ mice but this does not impact greatly on the ability to prime and recruit RSV-specific T cells that secrete IFN-γ and granzyme B into the lungs and airways. In summary, RSV-infected *Myd88/Trif/Mavs*^−/−^ mice are able to mount an RSV-specific T cell response and resist the virus despite a profound loss in innate immunity. These results indicate that mechanisms other than TLR, RLR and IL-1R signalling can induce adaptive immunity to RSV and, possibly, other RNA viruses.

## Results

### Airway inflammation after RSV infection is abrogated in *Myd88/Trif/Mavs* ^−/−^ mice

**Figure 1:**
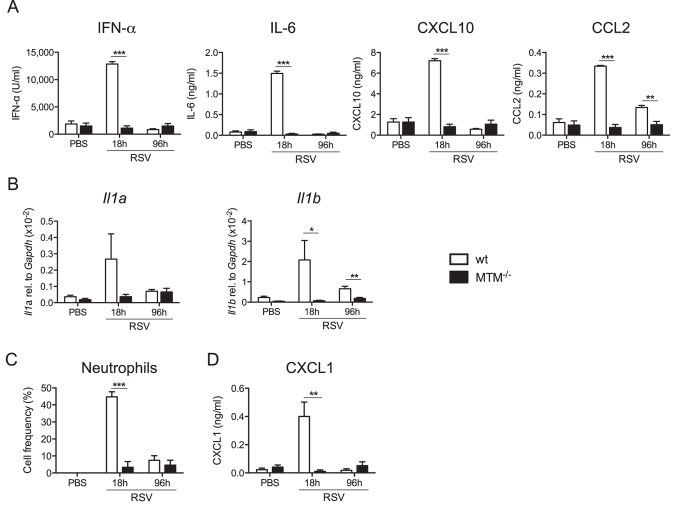
Mice lacking MyD88/TRIF and MAVS are unable to produce cytokines and chemokines during RSV infection. *Myd88/Trif/Mavs*^−/−^ (MTM^−/−^) and wt mice were intranasally infected with 2 × 10^6^ FFU of RSV or mock (PBS). At 18 h and 96 h post infection bronchoalveolar lavage (BAL) and lungs were collected. (**A**) Protein levels of IFN-α, IL-6, CXCL10 and CCL2 were determined in BAL fluid by ELISA. (**B**) Gene expression of *Il1a* and *Il1b* was determined by quantitative RT-PCR in lung tissue relative to *Gapdh*. (**C**) Percentage of neutrophils in the BAL was determined using differential cell counting of H&E stained cytospin slides. (**D**) Levels of CXCL1 (KC) in BAL fluid were quantified using ELISA. Data shown are representative of two experiments with 4–5 mice per group and depicted as mean ± SEM except in b where the data are pooled from two independent experiments with 3–5 mice per group and are depicted as mean ± SEM of 6–8 mice. ***p ≤ 0.001, **p ≤ 0.01, *p ≤ 0.05.

In order to delineate the role of TLRs, RLRs and IL-1/18R during RSV infection, *Myd88/Trif/Mavs*^−/−^ triply deficient mice (henceforth, MTM^−/−^) and control wild type (wt) mice (either C57BL/6 or littermate controls) were intranasally infected with RSV or mock infected with vehicle alone (PBS). At 18 h and 96 h post infection (p.i.), no IFN-α, IL-6, CXCL10 or CCL2 was detected in the bronchoalveolar lavage fluid (BAL) of infected MTM^−/−^ mice, in contrast to BAL from wt animals (Fig. 1A). These results are in line with previously published data using RSV-infected *Ifnar1*^−/−^, *Mavs*^−/−^ or *Myd88/Mavs*^−/−^mice^9^,^22^,^23^. Expression of IL-1α and IL-1β in the lungs was also undetected in MTM^−/−^ mice at any time point, in contrast to wt controls (Fig. 1B). Furthermore, very few neutrophils were recruited into the airways of MTM^−/−^ mice (Fig. 1C) consistent with the lack of production of the neutrophil chemoattractant CXCL1, which was tested both at protein and mRNA level (Fig. 1D and data not shown). Thus, signalling via TLRs and/or RLRs is necessary for the early pro-inflammatory cytokine and chemokine response to RSV infection and for ensuing infiltration of inflammatory cells.

### Alveolar macrophages from *Myd88/Trif/Mavs* ^−/−^ mice do not produce IFN-α, IL-6 and TNF-α after exposure to RSV

**Figure 2:**
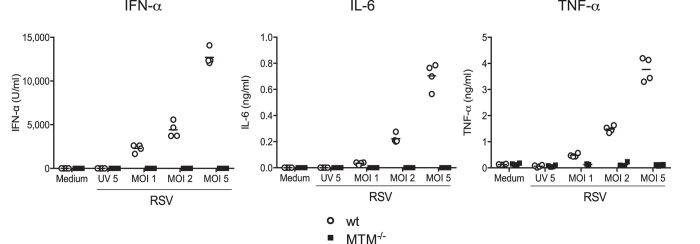
Alveolar macrophages from MTM^−/−^ mice do not produce cytokines after exposure to RSV *ex vivo*. Primary AMs from MTM^−/−^ and wt mice were exposed to medium, UV-inactivated RSV (UV 5; MOI of 5) or RSV (MOI of 1, 2 or 5). After 20 h, IFN-α, IL-6 and TNF-α levels were determined in the culture supernatant by ELISA. Data shown are representative of two experiments with four individual cultures per condition. The horizontal line represents the mean for each group.

Alveolar macrophages (AMs) are one of the first cell types encountering pathogens in the lower airways and are crucial to initiate early immune responses to RSV^9^. Primary wt AMs exposed *ex vivo* to RSV at different multiplicities of infection (MOI) produced IFN-α, IL-6 and TNF-α (Fig. 2). In contrast, no production was detected from AMs isolated from MTM^−/−^ mice (Fig. 2). These findings indicate that AMs rely on TLRs and/or RLRs signalling to respond to RSV, confirming our previous observation that RLR signalling is a dominant pathway in the innate immune response of these cells to RSV^9^.

### *Myd88/Trif/Mavs* ^−/−^ mice display impaired viral control and enhanced weight loss

**Figure 3:**
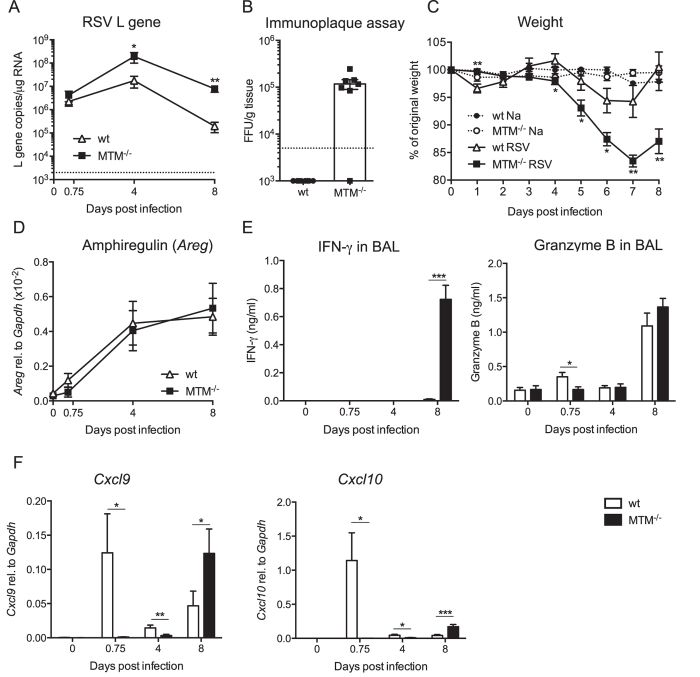
MTM^−/−^ mice show higher viral load, increased weight loss and IFN-γ and granzyme B production after RSV infection. MTM^−/−^ and wt mice were infected intranasally with 2 × 10^6^ FFU of RSV or mock (PBS). 0 h represents mock infected mice. (**A**) Levels of RSV L gene RNA in lung tissue were determined using quantitative RT-PCR at different time points after RSV infection. Copy numbers were determined using a plasmid standard and the results were normalised to levels of *Gapdh*. Data are shown as mean ± SEM of 6–13 mice per group, pooled data from two-three individual experiments with 3–5 mice per group in each experiment. The dotted line represents the detection limit. (**B**) Enumeration of infectious particles in lung tissue of wt and MTM^−/−^ mice using an immunoplaque assay on day 4 post infection. The dotted line represents the detection limit. Each dot represents an individual mouse and data are shown as mean ± SEM, pooled from two individual experiments with 3–4 mice per group in each experiment. (**C**) Weights of infected and non-infected wt and MTM^−/−^ mice were monitored daily and plotted as a percentage of weight on the day of infection (day 0). Data are shown as mean ± SEM of 10–16 mice per group, pooled from three-four individual experiments with 4–5 mice per group in each experiment. (**D**) Amphiregulin gene expression levels in lung tissue were quantified using quantitative RT-PCR and gene expression relative to *Gapdh* was calculated. Data are shown as mean ± SEM and are pooled from two individual experiments with 4–5 mice per group in each experiment. (**E**) Levels of IFN-γ and granzyme B detected in BAL fluid from PBS inoculated (0 h) or RSV-infected mice at different time points using ELISA. Data are shown as mean ± SEM of 7–13 per group, pooled from two-three individual experiments with 3–5 mice per group in each experiment. (**F**) CXCL9 and CXCL10 gene expression levels in lung tissue were quantified using quantitative RT-PCR and gene expression relative to *Gapdh* was calculated. Data are shown as mean ± SEM of 7–13 mice per group, pooled from two-three individual experiments with 3–5 mice per group in each experiment. ***p ≤ 0.001, **p ≤ 0.01, *p ≤ 0.05.

To assess the impact of the lack of MyD88/TRIF/MAVS signalling on the course of infection, RSV replication in the lung was determined (Fig. 3A,B). MTM^−/−^ mice displayed a much higher lung viral load than wt controls as measured by RSV L gene copies in lung tissue at day 4 and day 8 p.i.. As C57BL/6 is a relatively resistant mouse strain to RSV infection, isolating infectious virus from the lungs of these animals is not normally possible and they register as uninfected by immunoplaque assay where infected cells are stained positive for DAB (Fig. 3B). In contrast, lungs from MTM^−/−^ mice contained detectable infectious virus at day 4 p.i. using the same assay (Fig. 3B). In addition, the MTM^−/−^ mice lost significantly more weight than wt mice (17% versus 5% weight loss on day 7 p.i.; Fig. 3C). Nevertheless, from mice in a separate experiment, not sacrificed for analysis, it was clear that both wt and MTM^−/−^ mice survived the infection equally (data not shown). We tested whether loss of signalling through MyD88/TRIF/MAVS would influence the expression of the epidermal growth factor, amphiregulin, which is associated with epithelial repair^24^. Interestingly, MyD88/TRIF/MAVS did not regulate the gene expression of amphiregulin as it increased comparably in both wt and MTM^−/−^ mouse strains during the course of RSV infection (Fig. 3D). We conclude that mice deficient in MyD88/TRIF/MAVS display increased susceptibility to RSV infection, with higher virus titres and increased weight loss.

### IFN-γ, granzyme B, *Cxcl9* and *Cxcl10* are detectable at day 8 post RSV infection in the absence of MyD88/TRIF/MAVS signalling

To assess possible mechanisms of MyD88/TRIF/MAVS-independent resistance to RSV, we looked for indicators of antiviral activity and measured levels of IFN-γ and granzyme B in the airways (BAL fluid) at different time points after infection. IFN-γ was undetectable until day 8 p.i. in either MTM^−/−^ mice or wt mice. Notably, at that time point much higher BAL concentrations of IFN-γ were detected in MTM^−/−^ mice compared to wt mice (Fig. 3E). Granzyme B was detected in wt mice but not MTM^−/−^ mice at 18 h p.i. and reached similar levels in the two strains at day 8 p.i. (Fig. 3E). These data might suggest that an early source of granzyme B is missing in MTM^−/−^ mice, consistent with the defect in innate immunity in that strain, but is compensated at later stages by another source, most likely T cells. Indeed, lung recruitment of T cells reaches its peak at day 8 post RSV infection^25^ when they represent the main source of IFN-γ and granzyme B^26^. Consistent with the notion that lung T cell recruitment is functional in the absence of MyD88/TRIF/MAVS signalling, expression of the T cell chemoattractants CXCL9 and CXCL10^27^ was strongly induced in MTM^−/−^ mice at day 8 p.i., reaching even higher levels than in wt controls (Fig. 3F). Interestingly, MTM^−/−^ mice lacked an earlier peak of chemokine expression seen at 18 h p.i. in wt animals (Fig. 3F) again an indication that MTM^−/−^ mice might have a defect in recruitment of innate cells. Together, these data suggest that MyD88/TRIF/MAVS might be dispensable for a T cell response against RSV.

### T cells are recruited to the airways and lungs of *Myd88/Trif/Mavs* ^−/−^ mice after RSV infection

**Figure 4:**
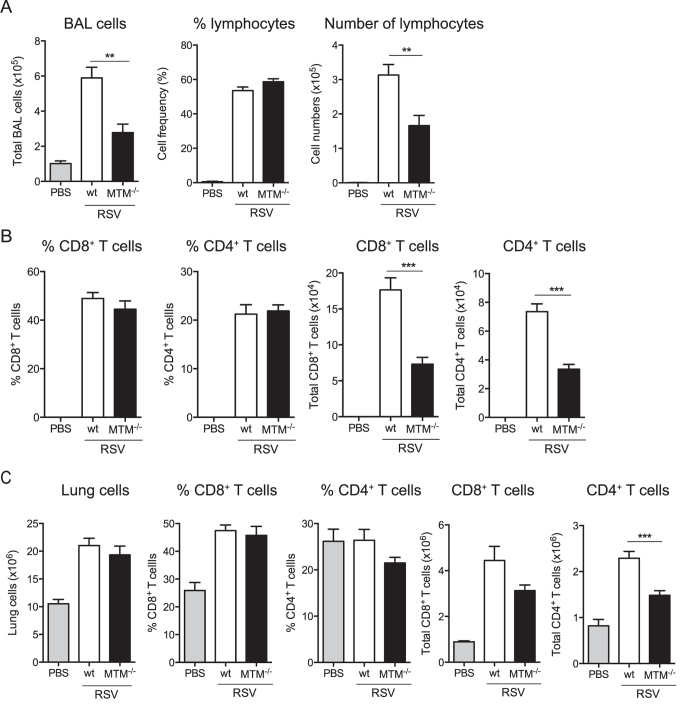
T cells are recruited to the airways and lungs of MTM^−/−^ mice after RSV infection. MTM^−/−^ and wt mice were intranasally administrated PBS or infected with 2 × 10^6^ FFU of RSV and at day 8 post infection BAL and lungs were analysed. (**A**) Total number of BAL cells was quantified and the frequency and quantity of lymphocytes were determined using differential cell counting of H&E stained cytospin slides. (**B**) Frequencies and absolute numbers of CD8^+^ and CD4^+^ T cells in the BAL were determined by flow cytometry on day 8 after PBS administration or RSV infection. (**C**) Total cells and frequencies and numbers of CD8^+^ and CD4^+^ T cells were quantified in the lungs of wt and MTM^−/−^ mice at day 8 p.i. using flow cytometry. Data are shown as mean ± SEM of 7–9 mice per group, pooled from two individual experiments. ***p ≤ 0.001, **p ≤ 0.01.

To test the above hypothesis, airways and lungs of MTM^−/−^ mice were analysed on day 8 p.i. for the composition of the immune infiltrate. The frequencies of lymphocytes (determined by differential counting of cytospin slides) and CD4^+^ and CD8^+^ T cells (determined by flow cytometry, gating strategy shown in Fig. S1A) in the BAL fluid were not different between MTM^−/−^ and wt control mice (Fig. 4A,B). However, the total number of cells in BAL was lower in MTM^−/−^ mice compared to wt mice (Fig. 4A) leading to a net reduction in CD4^+^ and CD8^+^ T cell numbers (Fig. 4A,B). Similar results were obtained when analysing lung tissue (Fig. 4C). Altogether, the defect in innate immune responses in the absence of MyD88/TRIF/MAVS did not hinder the T cell recruitment into the infected lungs and airways albeit MTM^−/−^ mice showed lower levels of T cells than wt mice.

### MTM^−/−^ mice display normal frequencies of RSV-specific CD8^+^ T cells

**Figure 5:**
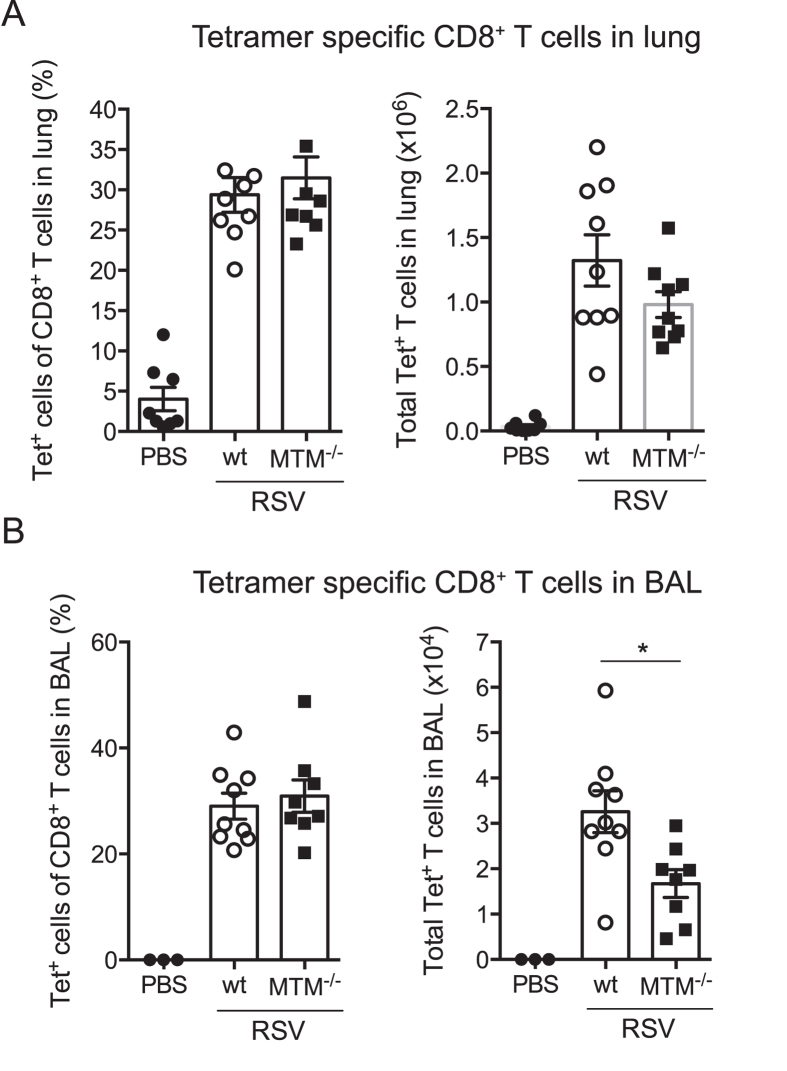
RSV-specific CD8^+^ T cells are recruited to the airways and lungs of RSV-infected MTM^−/−^ mice. MTM^−/−^ and wt mice were intranasally administered PBS or infected with 2 × 10^6^ FFU RSV. At day 8 post infection, lung and BAL cells were stained with MHC class I tetramer complexes specific for the RSV epitope H-2D^b^M_187-195_ and analysed by flow cytometry. Quantification of the percentages and total number of RSV H-2D^b^M_187-195_ specific CD8^+^ T cells in (**A**) lung tissue and (**B**) BAL. Each dot represents an individual mouse and data are shown as mean ± SEM of 5–9 mice per group, pooled from two individual experiments. *p ≤ 0.05.

To evaluate the RSV-specific T cell response, CD8^+^ T cells specific for the immunodominant epitope derived from the RSV M protein were quantified in lung and BAL on day 8 post RSV infection using MHC class I tetramer- M_187-195_ complexes (Fig. 5A,B; gating strategy shown in Fig. S1B). Frequencies of tetramer^+^ cells were similar between wt and MTM^−/−^ mice, with around 30% of the CD8^+^ T cells staining positive for RSV M tetramers (Fig. 5A,B). However, the total number of RSV-specific CD8^+^ T cells was lower in the BAL but not the lungs of MTM^−/−^ mice compared to wt mice (Fig. 5B), suggesting that MyD88, TRIF and/or MAVS signals might influence the extravasation of T cells into the airways. In sum, RSV-specific T cells can be elicited in mice deficient in MyD88/TRIF/MAVS.

## Discussion

DCs are activated in tissues by PRR and cytokine receptor signals and migrate to draining lymph nodes where they prime T cells. Many PRRs have been shown to recognise RSV but which are necessary to initiate virus-specific T cell responses is unclear. Here, we show that mice lacking the ability to signal via TLRs, RLRs, IL-1R or IL-18R (*Myd88/Trif/Mavs*^−/−^ mice) can still elicit RSV-specific T cells and their recruitment to the lung and airways. Our data indicate the existence of mechanisms for coupling detection of RSV infection to adaptive immunity that are not predicted from current knowledge in the field.

It is not clear how DCs get activated in the lungs of MTM^−/−^ mice during RSV infection in the absence of PRR signals. It has been suggested that, during influenza virus infection, CD8^+^ T cell priming depends on IL-1R rather than PRR signalling in DCs^13^. As MyD88 is required for IL-1R and IL-18R signaling^14^, this mean of activating DCs is not operative in our model. Consistent with that notion, we were also unable to detect any IL-1α or IL-1β in the lungs of MTM^−/−^ mice during RSV infection. Another possibility is that DCs get activated by products derived from dying cells^28^. DNGR-1 is a receptor that can couple detection of dead cells to CD8^+^ T cell priming^29^, even though it is not, technically, a DC-activating receptor^30^,^31^. However, we have recently shown that DNGR-1 is dispensable for T cell responses to RSV in mice^32^ although it could become non-redundant in the MTM^−/−^ mice, when activation of DCs via PAMPs is absent. Other dead cell-derived mediators that could promote DC activation include heat shock proteins, HMGB1, uric acid, and ATP^28^. An interesting candidate is DNA released from dead cells, which has been implicated in the adjuvant effects of alum^33^ and would trigger the cGAS/STING pathway normally involved in immunity to DNA viruses^34^. To what extent DNA sensing might impact the response to cytopathic RNA viruses is an interesting area for future research.

Underscoring the importance of PRRs in RSV infection, RSV bronchiolitis has been associated with polymorphism in genes encoding PRRs or their signalling pathways^35^,^36^,^37^,^38^,^39^. Here, we describe for the first time the impact of global loss of TLR and RLR signalling in experimental RSV infection. Consistent with a key role for PRRs in antiviral immunity, MTM^−/−^ mice showed markedly reduced innate responses with a deficit in production of pro-inflammatory cytokines or chemokines early post-infection. No infiltration by neutrophils was detected in the lungs of MTM^−/−^ mice and these mice were defective in restricting viral replication. NK cell responses were not directly evaluated in this study but our previous data showed that *Mavs*^−/−^ mice fail to recruit NK cells into the lungs during RSV infection^9^. Despite this early failure to contain RSV infection, MTM^−/−^ mice did not succumb to the virus as previously shown in STAT1^−/−^ mice^40^. This is most probably due to their capacity to mount RSV-specific T cell responses, as demonstrated here. Such responses have previously been reported following RSV infection of *Mavs*^−/−^ and *Myd88/Mavs*^−/−^ mice^9^,^21^,^22^ although the impact of additional TRIF deficiency has not been evaluated until now. We suggest that T cells recruited to the lung and airways in the MTM^−/−^ mice are more actively restimulated due to the higher viral burden, resulting in enhanced IFN-γ production that ultimately contributes to viral clearance.

Furthermore, we found that amphiregulin is upregulated in the lungs of RSV infected wt and MTM^−/−^ mice. Amphiregulin is a member of the epidermal growth factor family important for maintaining lung epithelial cell barrier function^24^,^41^. This and other growth factors might help to maintain the epithelial barrier during the innate phase of the response until T cells can be recruited. Interestingly, like T cell responses and the T cell recruiting chemokines CXCL9 and CXCL10 on day 8 p.i., amphiregulin was induced independently of MyD88, TRIF and MAVS signalling. Thus, signs of tissue repair and adaptive immune responses to RSV can occur independently of known classical PRR pathways implicated in the recognition of RNA viruses. It will be important to elucidate the mechanisms underlying this phenomenon as they might have an impact on future vaccine designs and therapeutics.

## Methods

### Mice

*Myd88/Trif*^−/−^ and *Mavs*^−/−^ mice (obtained from S. Akira, Japan^42^,^43^,^44^) were screened to ensure the genotype was maintained and were crossed in-house to generate *Myd88/Trif*^*−*^*/Mavs*^−/−^ mice. Littermates or C57BL/6 mice (purchased from Charles River or Harlan, UK) were used as controls. No difference between littermates and C57BL/6 controls were detected for any of the assays performed. All mice were bred and maintained in pathogen-free conditions and gender and age-matched mice aged 8–14 weeks were used for each experiment. All animal experiments were performed in accordance with the Animal Welfare and Ethical Review Board (AWERB) within Imperial College London and the ARRIVE guidelines and were approved by the UK Home Office in accordance with the Animals (Scientific Procedures) Act 1986.

### Cell isolation and processing

Mice were sacrificed using a fatal dose of pentobarbital injected intraperitonially (i.p.) according to UK home office guidelines. Bronchoalveolar lavage (BAL) and lungs were obtained as described previously^32^. Briefly, BAL was collected by flushing the lungs three times with 1 ml of PBS/0.5 mM EDTA (Life Technologies). Lung lobes were collected and digested with Collagenase D (1 mg/ml, Roche) and DNase I (30 μg/ml; Sigma-Aldrich) using a gentleMACs cell dissociator (Miltenyi Biotech) according to the manufacturer’s protocol and incubated at 37 °C for 30 min. Red blood cells were lysed by treating lung cells with ACK lysing buffer and lung cells were passed through 100 μM cell strainers to create single-cell suspensions. Total cell counts were determined using hemocytometer slides and dead cells were excluded by Trypan blue staining (Sigma-Aldrich). To determine the cellular composition in the BAL, cells were transferred onto a microscope slide (Thermo Scientific, UK) using a Shandon Cytospin 3 Centrifuge and slides were stained with hematoxylin and eosin (H&E; Reagena, Gamidor, UK). Cells were categorised as macrophages, lymphocytes or neutrophils based on their morphology and size under a light microscope.

For *ex vivo* stimulation of AMs, AMs were collected by bronchoalveolar lavage through flushing the lungs as above repeated twice and AMs from several mice were pooled. The purity of the AMs was >98%. Collected cells were incubated in a flat-bottom 96-well plate (1.25 × 10^5^ cells/well) in complete DMEM (cDMEM; supplemented with 10% fetal bovine serum, 2 mM L-glutamine, 100 U/ml penicillin and 100 μg/ml streptomycin) for 3 h. After washing, the adherent cells were exposed to cDMEM (medium) or various MOIs of RSV or UV-inactivated RSV for 20 h. Inactivation was performed by exposing virus to UV light for 2 min (UV-RSV) in a CX-2000 UV cross-linker (UVP).

### Virus and infection

Plaque-purified human RSV (originally A2 strain from the ATCC, US) was grown in HEp-2 cells^45^. For infection, mice were lightly anesthetised and installed intranasally (i.n.) with 2 × 10^6^ focus-forming units (FFU) of RSV in 100 μl. As control, intranasal administration of 100 μl PBS was given to naïve mice. Intranasal inoculation with PBS or HEp-2 cell supernatant does not change BAL cell numbers or cell composition compared to naïve mice (data not shown).

RSV titre was assessed in lungs 4 days post RSV infection using an immunoplaque assay (optimized from^26^). Briefly, lung homogenate was titrated on HEp-2 cell monolayers in 96-well, flat-bottom plates and incubated for 2 h on a shaker. Then, pyruvate-free DMEM containing 2% FCS was added and the plate was further incubated for 20 h before fixed with methanol and incubated with biotin-conjugated goat anti-RSV antibody (Biogenesis, United Kingdom). Infected cells were detected using Streptavidin (Sigma) and DAB substrate (Diaminobezindine tetrahydrochloride), enumerated by light microscopy and used to calculate titre as focus forming units (FFU).

### Flow cytometry

For staining, cells were first incubated for 30 min with fixable live-dead Aqua dye (Invitrogen), followed by incubation for 20 min with a purified rat IgG_2b_ anti-mouse CD16/CD32 receptor antibody (BD Bioscience) to block Fc receptors. Cells were then stained with fluorochrome-conjugated antibodies against CD3 (clone 145–2C11, PE-Cy7), CD4 (clone GK1.5, allophycocyanin-H7 or allophycocyanin-Cy7), CD8α (clone 53–6.7, AlexaFluor700) and CD19 (clone 1D3, FITC, eBioscience) in FACS staining buffer containing 1% BSA, 5 mM EDTA and 0.05% NaN_3_ for 25 min at 4 °C. All antibodies were purchased from BD Bioscience unless otherwise stated. For tetramer staining, cells were after incubation with fixable live-dead Aqua dye (Invitrogen) and Fc block, stained with Alexa Fluor 647-conjugated M_187-195_ tetramers (H-2D^b^/NAITNAKII), obtained from the NIH Tetramer Core Facility (Emory University Atlanta, GA, USA), for 30 min in the dark at RT. Surface staining was then performed as above. Afterwards, cells were fixed for 30 min in BD Cytofix/Cytoperm fixation buffer at 4 °C, washed and analysed on the flow cytometer. Samples were measured on a standard Becton Dickinson LSRII equipped with 50 mW 405 nm, 50 mW 488 nm, 20 mW 633 nm lasers and a ND1.0 filter in front of the FSC photodiode, or measured on a standard Becton Dickinson FortessaLSR equipped with 50 mW 405 nm, 50 mW 488 nm, 50 mW 561 nm, 20 mW 633 nm lasers and a ND1.0 filter in front of the FSC photodiode. Acquisition was set to record 250,000 live singlet cells. Data were analysed with FlowJo software (TreeStar).

### RNA isolation and qPCR

RNA extraction from lung tissue was performed using Trizol (Invitrogen) according to manufacturer’s instructions. One microgram of RNA was reverse-transcribed using a High Capacity RNA-to-cDNA kit according to manufacturer’s instructions (Applied Biosystems). To quantify mRNA levels in lung tissue quantitative RT-PCR was performed. RT-PCR reactions for *Areg* (Amphiregulin)*, Il1a, Il1b, Cxcl9, Cxcl10* (Applied Biosystems) and RSV L gene^23^ were performed using the Quantitect Probe PCR Master Mix (Qiagen) and the 7500 Fast Real-time PCR System (Applied Biosystems). For absolute quantification of RSV L gene, the exact number of copies of the gene was calculated using a plasmid DNA standard curve and then normalised to levels of *Gapdh*, a housekeeping gene (Applied Biosystems). For relative quantification, the expression of *Areg, Il1a, Il1b, Cxcl9 and Cxcl10* was expressed relatively to the expression of *Gapdh*. First, the ΔCt (Ct = cycle threshold) between the target gene and *Gapdh* for each sample was calculated, following the calculation of 2^−ΔCt^. Analysis was performed using 7500 Fast System SDS Software (Applied Biosystems).

### Chemokine and Cytokine detection

The concentration of CXCL10, CCL2, CXCL1, IFN-γ, TNF-α and Granzyme B was measured using mouse DuoSet ELISA (R&D) according to manufacturer’s instructions. IL-6 was detected by ELISA using MP5–20F3 capture antibody and biotinylated MP5–32C11 detection antibody (both from BD Pharmingen). IFN-α was detected by ELISA as previously described^46^. Data were acquired on a SpectraMax Plus plate reader (Molecular Devices) and analysed using SoftMax software (version 5.2).

### Statistical analysis

Results are presented as mean ± SEM. Statistical analysis was performed using GraphPad Prism 6 (GraphPad Software Inc.). Two-group comparisons were performed using two-tailed, unpaired, Student’s *t* test. For all tests a value of P < 0.05 was considered as significant. *p < 0.05; **p < 0.01; ***p < 0.001.

## Additional Information

**How to cite this article**: Goritzka, M. *et al.* T cell responses are elicited against Respiratory Syncytial Virus in the absence of signalling through TLRs, RLRs and IL-1R/IL-18R. *Sci. Rep.* **5**, 18533; doi: 10.1038/srep18533 (2015).

## Supplementary Material

Supplementary Information
