# Supplementary material for: T cell responses are elicited against Respiratory Syncytial Virus in the absence of signalling through TLRs, RLRs and IL-1R/IL-18R
Source: Sci Rep. 2015 Dec 21;5:18533. doi: 10.1038/srep18533 (PMC4685246; doi:10.1038/srep18533)
Supplement: Supplementary Information [file srep18533-s1.pdf]

SUPPLEMENTAL DATA

T cell responses are elicited against Respiratory Syncytial Virus in the absence of signalling through TLRs, RLRs and IL-1R/IL-18R

Michelle Goritzka[1], Catherine Pereira[1], Spyridon Makris[1], Lydia R. Durant[1] and Cecilia Johansson*[1]

Figure S1. Flow cytometry gating strategy for T cells during RSV infection. A) Representative plots of non-debris (SSC-A vs FSC-A), singlet (FSC-W vs FSC-A and SSC-W vs SSC-A), live (Dead cells vs SSC-A) lung cells analyzed for expression of CD3 and CD19. $CD3^+$ cells were further gated on CD4 and CD8 expression. B) Representative plots of expression of M Tetramer and CD8 on $CD3^+$ $CD19^-$ lung cells from wt and $MTM^{-/-}$ mice examined on day 0 (PBS) and day 8 post RSV infection.

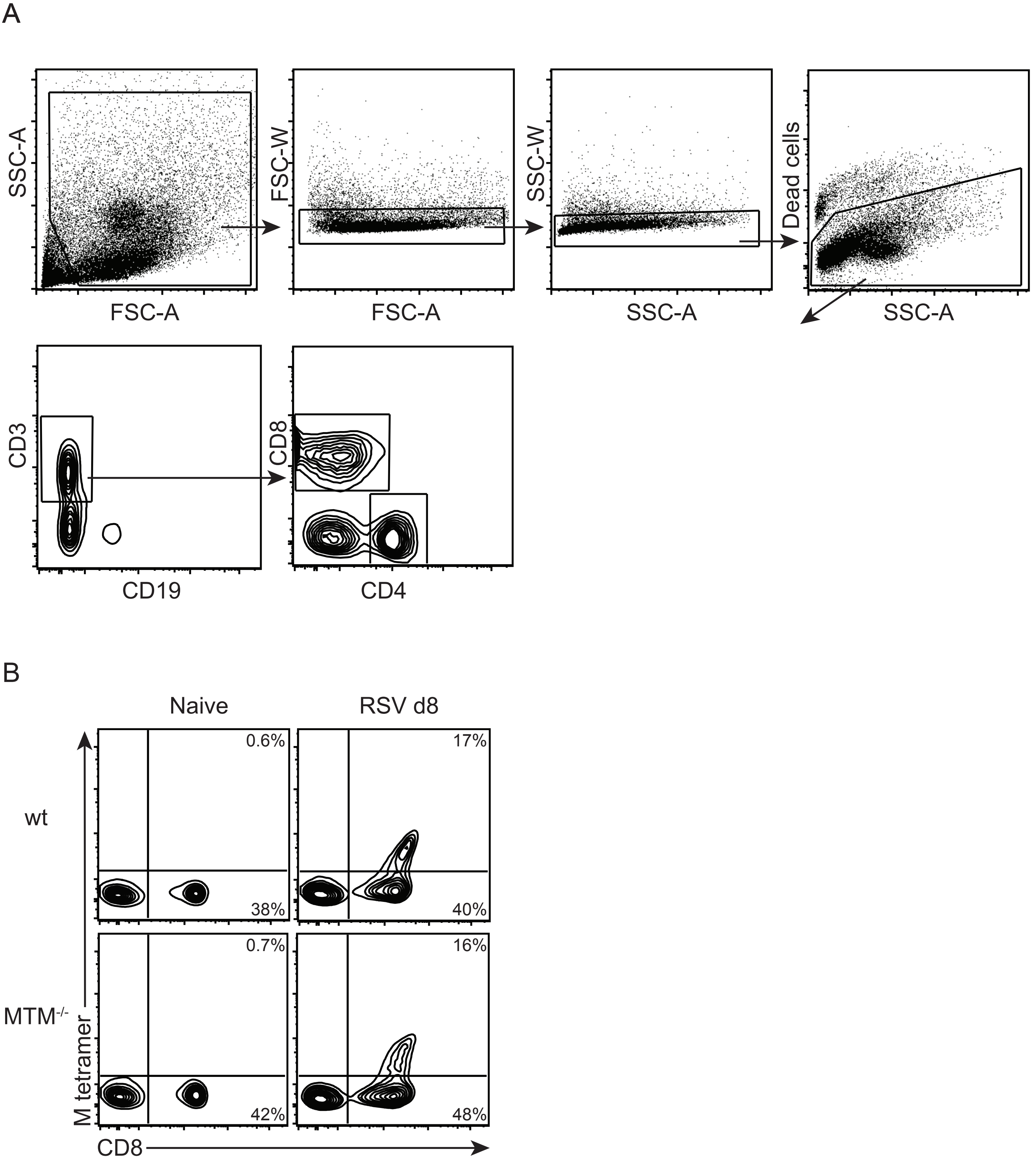

Figure S1. Goritzka et al
